# A patient with chest pain and a pulsating left main coronary artery

**DOI:** 10.1007/s12471-019-01324-w

**Published:** 2019-08-14

**Authors:** A. Y. Andreou, A. Karyou, A. Argyrou

**Affiliations:** 1 Department of Cardiology, Limassol General Hospital, Limassol, Cyprus; 2 University of Nicosia Medical School, Nicosia, Cyprus; 3 Department of Cardiology, Paphos General Hospital, Paphos, Cyprus

A 42-year-old female patient, a cigarette smoker with no known medical history, was referred for emergency coronary angiography because of acute coronary syndrome (ACS). She presented with sudden-onset chest pain associated with electrocardiographic evidence of ischaemia (Fig. 1a). Her blood pressure was 90/25 mm Hg. The right radial artery pulse was non-palpable. Thus we performed transfemoral coronary angiography, which showed no evidence of atherosclerosis but a smooth-bordered ostial and mid-shaft left main coronary artery (LMCA) stenosis with dynamic compression and almost complete lumen obliteration during diastole (Fig. 1b, c; Electronic Supplementary Material, Video 1). We suspected proximal aortic dissection (AD), which was confirmed by emergency echocardiography (Electronic Supplementary Material, Video 2). Indeed, LMCA pulsation was due to retrograde extension of the aortic false lumen into the LMCA causing diastolic compression of the true coronary lumen. The patient underwent a successful AD repair with Bentall’s procedure and hemi-arch replacement and patch repair of the dissected LMCA.

**Fig. 1:**
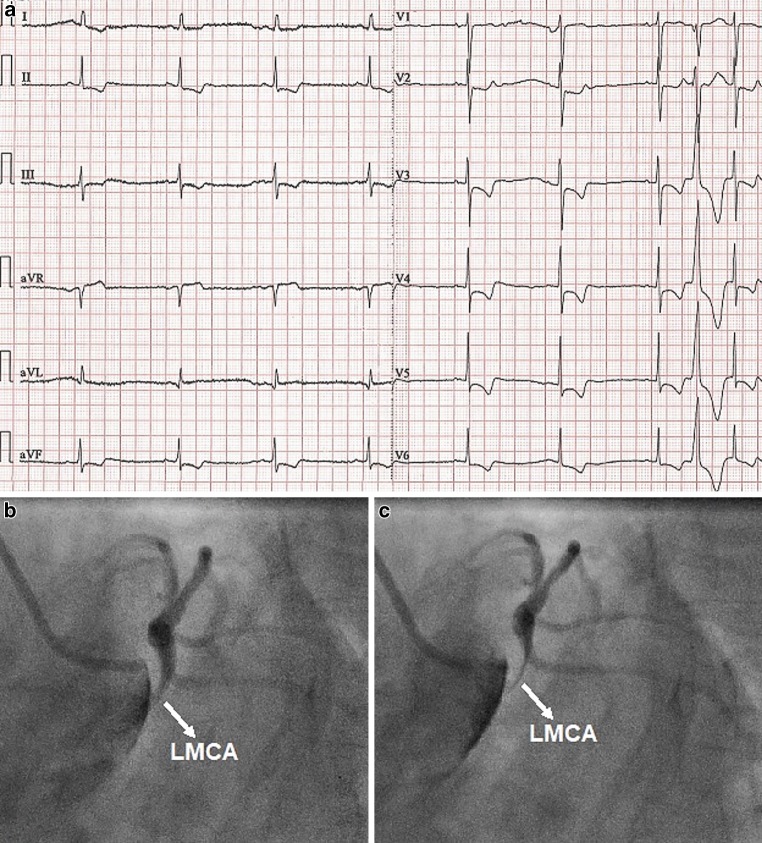
**a** Twelve-lead electrocardiogram on admission depicting ST-segment depression and negative T wave in leads II, aVF, III and V2-V6 and ST-segment elevation in lead aVR. **b** Non-selective left coronary artery (LCA) angiogram (systolic frame) in the left anterior oblique (LAO) caudal projection depicting a narrowed left main coronary artery (*LMCA*) due to an obstructing false lumen that extended from the aorta. **c** Non-selective LCA angiogram in the LAO caudal projection depicting a slit-like lumen of the LMCA in diastole due to compression by the false lumen

Acute proximal AD is complicated by retrograde dissection into either or both coronary ostia in 5.7–15% of cases [1, 2]; misdiagnosis with ACS may have a fatal outcome [3]. Dynamic LMCA lumen compromise during diastole is highlighted herein as a subtle and rare angiographic finding that should alert the interventional cardiologist to possible proximal AD extending into the LMCA.

## Caption Electronic Supplementary Material

*Video 1* Non-selective left coronary artery angiography showing dynamic left main coronary artery lumen compromise during diastole

*Video 2* Echocardiographic image. Parasternal long-axis view showing a dilated aortic root harbouring a dissection flap

## References

[1] Neri E, Toscano T, Papalia U (2001). Proximal aortic dissection with coronary malperfusion: presentation, management, and outcome. J Thorac Cardiovasc Surg.

[2] Imoto K, Uchida K, Karube N (2013). Risk analysis and improvement of strategies in patients who have acute type A aortic dissection with coronary artery dissection. Eur J Cardiothorac Surg.

[3] Kawano H, Tomichi Y, Fukae S, Koide Y, Toda G, Yano K (2006). Aortic dissection associated with acute myocardial infarction and stroke found at autopsy. Intern Med.

